# Complete Nucleotide Sequence and Analysis of Two Conjugative Broad Host Range Plasmids from a Marine Microbial Biofilm

**DOI:** 10.1371/journal.pone.0092321

**Published:** 2014-03-19

**Authors:** Peter Norberg, Maria Bergström, Malte Hermansson

**Affiliations:** 1 Department of Infectious Diseases, University of Gothenburg, Göteborg, Sweden; 2 Department of Chemistry and Molecular Biology, Microbiology, University of Gothenburg, Göteborg, Sweden; University of Manchester, United Kingdom

## Abstract

The complete nucleotide sequence of plasmids pMCBF1 and pMCBF6 was determined and analyzed. pMCBF1 and pMCBF6 form a novel clade within the IncP-1 plasmid family designated IncP-1 ς. The plasmids were exogenously isolated earlier from a marine biofilm. pMCBF1 (62 689 base pairs; bp) and pMCBF6 (66 729 bp) have identical backbones, but differ in their mercury resistance transposons. pMCBF1 carries Tn*5053* and pMCBF6 carries Tn*5058*. Both are flanked by 5 bp direct repeats, typical of replicative transposition. Both insertions are in the vicinity of a resolvase gene in the backbone, supporting the idea that both transposons are “res-site hunters” that preferably insert close to and use external resolvase functions. The similarity of the backbones indicates recent insertion of the two transposons and the ongoing dynamics of plasmid evolution in marine biofilms. Both plasmids also carry the insertion sequence IS*Pst1*, albeit without flanking repeats. IS*Ps1*is located in an unusual site within the control region of the plasmid. In contrast to most known IncP-1 plasmids the pMCBF1/pMCBF6 backbone has no insert between the replication initiation gene (*trfA*) and the vegetative replication origin (*oriV*). One pMCBF1/pMCBF6 block of about 2.5 kilo bases (kb) has no similarity with known sequences in the databases. Furthermore, insertion of three genes with similarity to the multidrug efflux pump operon *mexEF* and a gene from the NodT family of the tripartite multi-drug resistance-nodulation-division (RND) system in *Pseudomonas aeruginosa* was found. They do not seem to confer antibiotic resistance to the hosts of pMCBF1/pMCBF6, but the presence of RND on promiscuous plasmids may have serious implications for the spread of antibiotic multi-resistance.

## Introduction

Plasmids are independent from the bacterial host chromosome and conjugative broad-host range (BHR) plasmids can transfer between many different species in a bacterial community. Thus, plasmids are a particularly fluid part of the bacterial genome and one that may provide the cell with new traits that allow adaptation to selection forces, especially to transient environmental changes and challenges. Plasmids are likely to evolve differently in different bacterial backgrounds [1] and might also be influenced by environmental conditions [2]. Thus analysis of plasmids from different environments is important for our understanding of these important mobile elements (MGE). There are a number of studies about plasmids in marine environments [3], [4], [5], [6], [7], [8], [9], [10], [11], as well as reports about the role of marine plasmids in antibiotic resistance [12], [13], [14], [15] and population dynamics [16], [17], for excellent reviews on this topic see [18], [19]. Still, compared with many other environments, plasmids from marine habitats are not well studied and fully sequenced plasmids with marine origins are underrepresented in the databases.

Using an exogenous isolation strategy, we have earlier isolated plasmids from marine bulk water, biofilms and the surface microlayer [20]. One of these plasmids, pMCBF1 isolated from a biofilm, was shown to have a BHR and transferred to many Gram-negative bacteria, including *Planctomyces maris* [21]. We also measured comparatively high transfer rates of pMCBF1 from a *P. putida* donor to indigenous bacteria, directly in seawater [4]. pMCBF1 was therefore an interesting BHR marine biofilm plasmid to characterize further. We determined the complete nucleotide sequences of pMCBF1 and the related pMCBF6, and a brief summary of the plasmids was presented earlier [1]. It was also shown that they form a novel clade within the promiscuous IncP-1 plasmid incompatibility group [1].

Since pMCBF1 and pMCBF6 are the only representatives of their IncP-1 clade, and except for pMLUA1, pMLU3 and pMUA4 [22], so far the only sequenced IncP-1 plasmids isolated from marine environments, it is interesting to analyze both their backbone genes and their accessory genes, and to compare these with other IncP-1 plasmids. Here we present a detailed analysis of pMCBF1 and pMCBF6.

## Materials and Methods

### Bacterial strains, plasmids and growth conditions

*P. putida* UWC1 [23] and *P. putida* KT2440 [24] with pMCBF1 and pMCBF6 were grown overnight at 26°C in Luria-Bertani media [25] with 10 g NaCl per liter and supplemented with HgCl_2_ at 17 mg per liter. *Escherichia coli* with pMCBF1 and pMCBF6 were grown overnight at 37°C in the same medium supplemented with ampicillin (50 mg/liter).

### Mercury and antibiotic resistance tests

To test for phenyl mercury resistance, *P. putida* KT2440 with either pMCBF1 or pMCBF6 were grown overnight and spread as lawns on LB plates. Discs that were dipped in solutions of phenyl mercury chloride (saturated solution in ether) were dried and placed on the lawns. Plates were inoculated overnight at 30°C after which the diameters of clearing zones around the discs were recorded. *P. putida* KT2440 without plasmids was used as controls. For the antibiotic resistance tests susceptibility discs (OXOID, UK) were placed on bacterial lawns of *E. coli* HB101 [26] and CAG [27] with and without pMCBF1 and pMCBF6 and incubated in 37°C overnight and clearing zones recorded as above.

### Sequencing and sequence analysis

Sequencing of pMCBF1 and pMCBF6 was performed earlier in our laboratory using standard techniques [1]. Sequence alignments presented here were created using the eBioX program, the phylogenetic network was created using the SplitsTree program [28], and the similarity analysis was carried out using the SimPlot program [29]. The content of the genomes of the IncP-1 plasmids vary and all genes are not present in all plasmids [1]. The network constructed here was based on the largest segment that we could identify in which all genes were present in all analyzed plasmids (TraC to TraM).

### Nucleotide sequence accession numbers

The complete sequences from pMCBF1 and pMCBF6 are deposited with GeneBank CoreNucleotide (accession # AY950444 and EF107516, respectively).

## Results and Discussion

**Figure 1:**
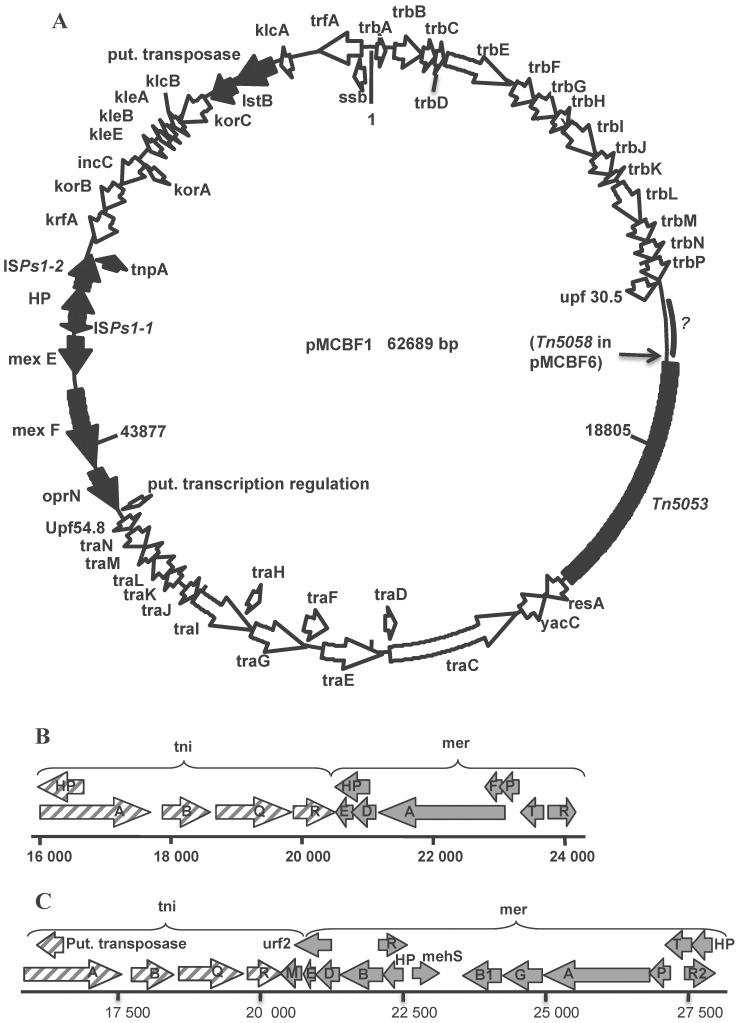
Genetic map of the IncP-1 ς plasmids pMCBF1 and pMCBF6. The backbones of the two plasmids are identical and differ only with regard to inserted transposons. Panel (A) shows pMCBF1 with Tn*5053*. Coding regions are shown by arrows, indicating the direction of transcription. Unfilled arrows denote plasmid backbone genes, black arrows denote accessory genes. An arrow points to the insertion site of Tn*5058* in the pMCBF6 backbone. The section of the pMCBF1/pMCBF6 backbone that has a low similarity to other sequences in the databases is marked with a question mark. Panels B and C shows transposon Tn*5053* on pMCBF1 and Tn*5058* on pMCBF6, respectively. Striped arrows denote genes in the transposition module (tni) and gray arrows denote mercury resistance (mer) genes. HP means hypothetical proteins.

pMCBF1 and pMCBF6 represent the first fully sequenced IncP-1 plasmids from marine environments. Generally the difference between the IncP-1 plasmids sequenced so far lies in the various insertions of resistance and catabolic genes, often as part of MGEs, onto a more or less conserved backbone structure. pMCBF1 and pMCBF6 are obvious examples of this since they differ only in their mercury resistance (Hg^R^) transposons that have inserted on otherwise identical backbones (Fig. 1A). Furthermore, the similarity of pMCBF1 and pMCBF6 strongly indicates that the insertions of Tn*5053* in pMCBF1 and Tn*5058* in pMCBF6 are recent events.

### Plasmid backbone

**Figure 2:**
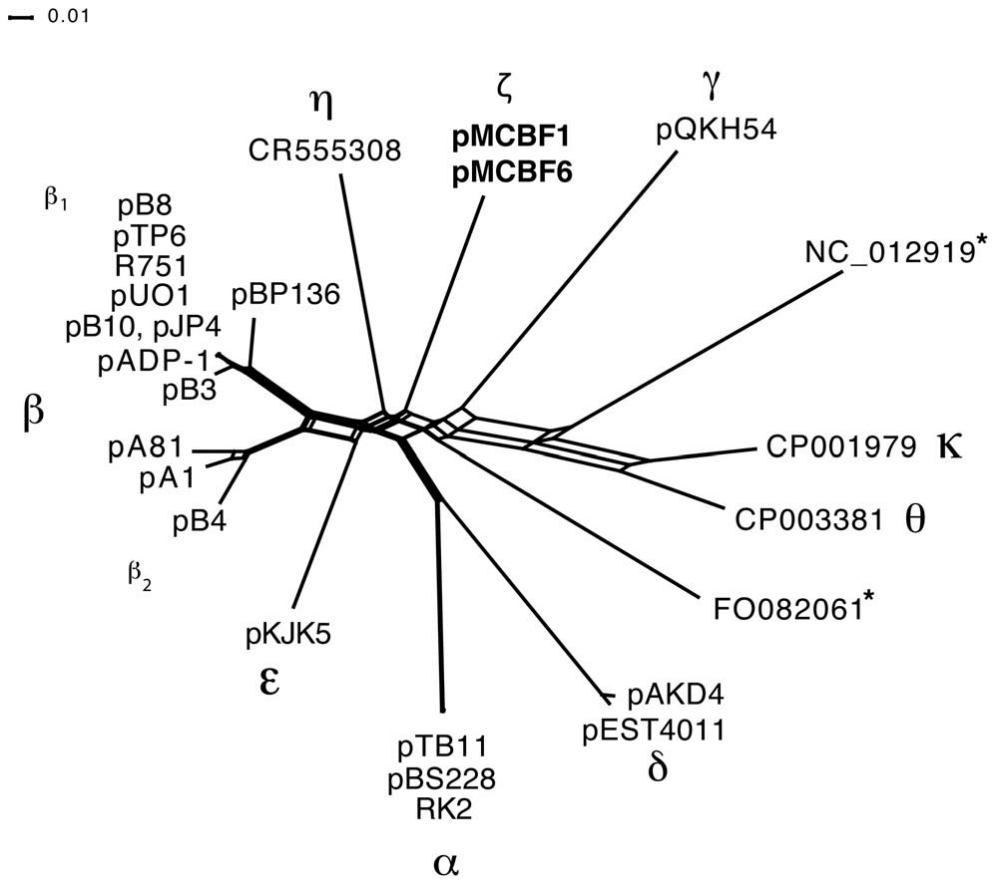
Phylogenetic network of clades of the IncP-1 plasmid family. The network is based on representative plasmids from each of the twelve previously described phylogenetic clades of the IncP-1 plasmid family. The network is based on the genetic segment harboring the *traC* - *traM* genes for all analyzed plasmids. The two plasmids pMCBF1 and pMCBF6 described in this study are highlighted in bold. Clades without designated names are marked with *. Previously described intra-clade recombinants [1] were not included in the analysis. The figure is updated from [1].

Although the focus of most previous studies has been on accessory genes and MGEs, it has been shown that also the IncP-1 plasmid backbones vary and can be divided into different evolutionary clades. Hitherto, twelve phylogenetic clades have been described for the IncP-1 plasmid group, which are designated α, β-1, β-2, γ, δ, ε, ς, κ, η, θ [1], [30], [31], [32], [33], [34], [35] and two unnamed groups (Fig. 2). The pMCBF1/pMCBF6 plasmids described here are so far the only described plasmids from the IncP-1 ς-clade.

As shown in Fig 2, the members of the IncP-1 plasmid backbone family form a star-phylogeny. Such topology is typical when there has been extensive recombination [36], [37], exponential population growth [38], [39], or if members of each clade have evolved in different distinct separated populations (i.e., long time of evolution since a common ancestor result in long external branches). It has previously been demonstrated that homologous recombination has contributed to the evolution of the IncP-1 backbones [1]. IncP-1 plasmids have also been found in different environments, such as soil, wastewater treatment plants, and in human pathogens and fish pathogens [35], and as described here in a marine biofilm. Furthermore, it was recently demonstrated that the IncP-1 plasmid backbone has evolved in, and genetically adapted to, vastly different host bacterial species [1]. It is thus likely that the IncP-1 backbone at least periodically has evolved in different separated populations, which may explain the diversity and the star-shaped phylogeny of this BHR plasmid family.

We have identified 44 open reading frames (ORFs) in the pMCBF1/pMCBF6 backbone. pMCBF1 has 24 ORFs and pMCBF6 28 ORFs that are related to accessory genes or functions such as transposons, various resistance functions, IS elements and multi-drug efflux systems. There are various genetic distances between the pMCBF1/pMCBF6 backbone genes and the corresponding genes in plasmids from the other clades, which is probably explained by the history of recombination [1]. As an example, most of the backbone genes such as replication, conjugation and stable maintenance genes share 58–95% similarity with genes from plasmids pADP-1 [40], pB4 [41], R751 [31] and pTSA [42] from the β clade, which is the most studied IncP-1 clade.

The origin of replication (*oriV*) of pMCBF1/pMCBF6 resembles other IncP-1 plasmids. The nine TrfA DNA binding sites (iterons) in the vicinity of the pMCBF1/pMCBF6 *oriV* have the 17 bp consensus sequence N(T/C/G)GCCCCTCA(A/T)(A/G/C)T(A/G)T(C/T)A, and have been conserved to some degree compared to both RK2 and R751. The R751 iterons by comparison have the sequence (A/C/G)NGCCCC(T/C)C A(A/T)(G/C)(T/G)GTCA. The pMCBF1/pMCBF6 iterons are arranged as eight direct repeats and one inverted repeat, similar to the iterons close to *oriV* in R751.

The TrfA protein is necessary for the replication of iteron containing theta replication plasmids (e.g. [43]) and *trfA* has been used as a base for plasmid classification [44]. We suggested earlier that pMCBF1 and pMCBF6 had replication functions that were different from known plasmid incompatibility groups based on lack of hybridization with the *inc/rep* Couturier probes [20]. However, the general lack of similarity of the Couturier probes with many environmentally isolated plasmids has been noted in several investigations [9], [18], [45], [46], [47]. We now know that the IncP-1 Couturier probe only detects the IncP-1 α clade [48]. In fact, the nucleotide similarity between pMCBF1/pMCBF6 and the Couturier IncP-1 probe is only about 60% and these plasmids could therefore not be targeted by the probe.

The pMCBF1/pMCBF6 backbone has no insert between the replication initiation gene *trfA* and the *oriV*. Except for the IncP-1 β2 plasmids pBP136 and pA1, most other IncP-1 plasmids have an insert, usually several kilobases (kb), that separates *trfA* and *oriV*.

Just as for the IncP-1 β plasmids, pMCBF1/pMCBF6 lack the *parABCDE* genes that play a key role in stability of IncP-1 α plasmids. Although the *parABCDE* genes may be absent from pMCBF1/pMCBF6, they apparently possess the *korA*-*incC*-*korB* locus that mediates accurate plasmid segregation. Surface exclusion systems also protect the resident plasmid from competition, by preventing new plasmids to enter the cell. pMCBF1/pMCBF6 carry the *trbK* homologue, which confers surface exclusion in plasmid RK2. We know from recombination analyses that pMCBF1/pMCBF6 have been involved in recombination events [1], which strongly suggest that these plasmids have been in the same host cell as other IncP-1 plasmids [1]. Thus, it seems that the surface exclusion systems of pMCBF1/pMCBF6 have a “leakiness” that allow recombination to occur.

One block of about 2.5 kb, between the inserted Hg^R^ transposons and the *trb* section (marked with a question mark in Fig.1) has a low similarity (73% identity) to *Methylophaga* sp. JAM7 [49].

### Accessory genes, transposons and insertion sequences

The DNA segment bearing the mercury resistance determinant on pMCBF1 (Fig. 1B) shows 99% nucleotide similarity to Tn*5053* [50]. There are five base pair direct repeats in the flanking plasmid DNA, which are typically formed after Tn*5053* transposition [50]. Interestingly, Tn*5053* in pMCBF1 is inserted close to the resolvase gene (*resA*) in the backbone (Fig. 1A) which supports the suggestion that Tn*5053* is a “resolvase-site-hunter” that insert in “hot-spots” associated with *res* sites and resolvase genes [51]. Minakhina *et al.* suggested that the plasmid resolvase may participate in the transposition event, but the exact mechanism is not clear [51]. If so, this is an interesting case in which the transposon is dependent on an external site-specific recombination system. The two inverted repeats (IR) that form the ends of Tn*5053* in pMCBF1 have 2 and 4 mismatches compared with other published sequences [52]. There are also 4 mismatches between the two IR of Tn*5053* in pMCBF1. However, this apparently did not stop transposition of Tn*5053* into pMCBF1. Tn*5053* belongs to a superfamily of diverse elements including retroviruses, phage Mu, insertion sequences of the IS*3* family, and transposons Tn*552*, Tn*7* and Tn*5058* [53]. They are found on plasmids within the IncP-1 group (pPUO1, pB11 and pMCBF1/pMCBF6) as well as on other plasmid groups, such as the *Enterobacter cloacae* plasmid pELC_A [54].

The DNA segment bearing the mercury resistance determinant on pMCBF6 (Fig. 1C) is highly similar to Tn*5058* in *Pseudomonas* sp. ED23-33 [55]. The length of both sequences are 12, 373 bp and the nucleotide similarity varies from 96 to 99% except for the first 257 bp where similarity is 84%, but the IR at the start of the element again has a perfect match with Tn*5058*. The element has 25 bp inverted repeats with 2 mismatches. The complex Tn*5058* is part of the Tn*5053* family (see above) [53], [56]. Judging from comparisons with the closest relatives of Tn*5058*, such as Tn*21*, Tn*501*, Tn*5053*, Tn*5041D*, Tn*5718* and others, it was suggested that Tn*5058* was formed by a complicated series of recombination events involving these elements [56]. Tn*5058* is also carried by other IncP-1 plasmids such as pIJB1, pWEC911 and pTP6. Interestingly, Tn*5058* has been detected in bacteria preserved for 15.000–40.000 years in permafrost grounds [56]. When compared to several Tn*5058* collected in modern days, there were high sequence similarities suggesting that these transposons are genetically stable over time. We compared the Tn*5058* in plasmid pMCBF6 with the Tn*5058* isolated from permafrost, using the sliding window protocol implemented in the SimPlot program. Our results support previous suggestions that the Tn*5058* is indeed well conserved and there is a high nucleotide identity between the transposon Tn5058 isolated here and the transposon Tn5058 isolated from permafrost in most regions. Least conserved was the gene *tniA* with a 200 bp region of only than 80% identity and *merB* and *merR* with regions of less than 95% identity between the Tn*5058* in pMCBF6 and the Tn*5058* isolated from permafrost. Tn*5058* has two copies of *merR, merB* and *merD*. Interestingly, it was suggested that the duplications in some of the *mer* genes in Tn*5041D* were the result of an integration, via homologous recombination, of a *mer* containing circular DNA structure [55]. The circular cassette was speculated to originate from an ancestral donor in which the *mer* genes were flanked by IS elements. The duplication of *mer* genes in Tn*5058* might also have been the result of a similar event [55]. Just as for Tn*5053* in pMCBF1, Tn*5058* is flanked by 5 base pair direct repeats in pMCBF6, which strongly indicate a transposition event [50], [52]. These flanking direct repeats are also seen when Tn*5058* is inserted in IncP-1 plasmid pIJB1. The insertion of Tn*5058* in pMCBF6 is only 63 bp from the site where Tn*5053* inserted in pMCBF1, which is close to the resolvase gene (*resA*) in the plasmid backbone. The insertion site further confirms that Tn*5058* is a “res-site hunter” [51].

The sequence data suggest that Tn*5053* confers mercury resistance towards inorganic mercury while Tn*5058* also carries resistance towards organo-mercury compounds. When the two plasmids were tested for phenyl mercury chloride (PhHg) resistance the diameter of the clearing zones around PhHg discs was only 6 mm for *P. putida* 2440(pMCBF6) but 13 mm for *P. putida* 2440(pMCBF1) and the plasmid-free *P. putida* 2440, confirming the PhHg resistance of pMCBF6 but not of pMCBF1.

A 2982 bp sequence between *kfrA* and *mexE* in both pMCBF1 and pMCBF6 shows 99% (nucleotide) similarity with insertion sequence IS*Ps1* (Fig. 1A). The 24 bp IR regions with 4 mismatches characteristic of IS*Ps1* [57] are found in pMCBF6/pMCBF1. IS*Ps1* is related to the IS*L3* family, but is larger than the usual IS*L3* elements which are 1300–1550 bp. IS*Ps1* has been found in several copies in *Pseudomonas stutzeri*, where it has inserted into, and inactivated, catabolic genes [57], and in *Yersinia ruckeri* plasmid pYR1 [58]. Interestingly, IS*Ps1* was also recently found on plasmid pAMEC615 from the marine *Alteromonas macleodii* isolated from the English Channel [59]. In *Pseudomonas stutzeri*, IS*Ps1* was flanked by eight bp direct repeats (DR), indicating a replicative transposition event [57], but no such flanking DR was found in any of pMCBF1/pMCBF6, pAMEC615 or pYR1. If DR were formed during a replicative transposition in these plasmids, they may have degenerated with time, perhaps because these sequences are not under selective pressure. Alternatively, the IS*Ps1*was inserted by some other recombination mechanism than replicative transposition. The insertion point of IS*Ps1* in pMCBF1/6, within the plasmid control region between *kfrA* and *upf54.8*, seems to be unique among IncP-1 plasmids, indicating that insertion points other than the ones between *tra* and *trb* regions and between the replication region and *oriV*, are possible.

A region containing a putative remnant of an insertion event is located between *klcA* and *klcB*. This region contains an ORF with closest similarity (74%) to a putative transposase, as well as an IstB homolog. IstB is associated with IS*21* family insertion sequences. The function of IstB is unknown, but it may assist in transposition [60]. We find no DR or IR as signs of a recent insertion. Insertions of transposons in the *klcA*/*klcB* region are seen also in IncP-1 plasmid RP1/RK2 [61].

Adjacent to IS*Ps1* is an inserted region that shows a high similarity to part of the recently sequenced plasmid pALIDE201 from *Alicycliphilus denitrificans* isolated from anaerobic sewer sludge [62]. Furthermore, three genes within this region show similarity (73–67%) to *mexEF* and a gene from the NodT family, which are part of tripartite multi-drug resistance-nodulation-division (RND) families. Such efflux systems are common on chromosomes of many Gram-negative bacteria [63], e.g. the *mexEF-oprN* in *Pseudomonas aeruginosa*. The IncP-1 β plasmid pB4 has a region with about 80% similarity to a RND efflux system in *P. denitrificans*, this was the first plasmid that was shown to carry a RND efflux system [41]. Adjacent to the RND region in pB4 a putative transposon terminus was found close to a Tn*3*-like transposase, and the authors speculated that the RND efflux system might have been transferred to pB4 from the chromosome of an unknown gram-negative bacterium by transpositional cointegrate formation [41]. Although IS*Ps1* is found adjacent to the *mex-*region in pMCBF6/pMCBF1, we do not know if this IS element was involved in the *mex* insertion. The MexEF-OprN system in *P. aeruginosa* confers resistance to aromatic hydrocarbons, fluoroquinolones, chloramphenicol, triclosan and trimethoprim [63]. Our experimental data shows that when tested in disk diffusion tests for antibiotic sensitivity on agar plates, *E. coli* with pMCBF1 or pMCBF6 did not confer a higher resistance to chloramphenicol, nalidixic acid or trimethoprim than plasmid free cells. Further studies are needed to define the possible function of MexEF-OprN in pMCBF1/pMCBF6. Our finding that plasmids from a marine biofilm carry a multi-drug efflux system shows that pB4 is not unique, and that many different efflux systems may potentially be borne by promiscuous plasmids. The possibly serious implications of multi-drug efflux systems on such plasmids and the effects this may have on the spread of multi-resistance to human pathogens have been discussed [41].

The majority of the well-known IncP-1 plasmids, and plasmids in general, originate from soil environments or from a clinical environment and other man-made systems, such as wastewater treatment plants [35]. pMCBF1/pMCBF6 are among the first IncP-1 plasmids that are sequenced from marine environments. Three IncP-1ε plasmids (pMLUA1, pMLUA3, pMLUA4) were recently isolated from the air-water interface from an estuary in Portugal [22], [64]. Analysis of these, and other plasmids from marine bacteria, has often revealed new features [7], [10], [11], [22]. Analysis of the pMCBF1/pMCBF6 plasmids also showed, among other things, that they form a new clade within the IncP-1 plasmid group.
